# Associations between psychological stress, eating, physical activity, sedentary behaviours and body weight among women: a longitudinal study

**DOI:** 10.1186/1471-2458-13-828

**Published:** 2013-09-11

**Authors:** Jennifer Mouchacca, Gavin R Abbott, Kylie Ball

**Affiliations:** 1 Centre for Physical Activity and Nutrition Research, School of Exercise and Nutrition Sciences, Deakin University, 221 Burwood Highway, Burwood, VIC 3125, Australia

**Keywords:** Psychological stress, Eating, Physical activity, Sedentary behaviours, Body weight, Regression analyses

## Abstract

**Background:**

There is an increased risk of obesity amongst socioeconomically disadvantaged populations and emerging evidence suggests that psychological stress may be a key factor in this relationship. This paper reports the results of cross-sectional and longitudinal analyses of relationships between perceived stress, weight and weight-related behaviours in a cohort of socioeconomically disadvantaged women.

**Methods:**

This study used baseline and follow-up self-report survey data from the Resilience for Eating and Activity Despite Inequality study, comprising a cohort of 1382 women aged 18 to 46 years from 80 of the most socioeconomically disadvantaged neighbourhoods in Victoria, Australia. Women reported their height (baseline only), weight, sociodemographic characteristics, perceived stress, leisure-time physical activity, sedentary and dietary behaviours at baseline and three-year follow-up. Linear and multinomial logistic regression were used to examine cross-sectional and longitudinal associations between stress (predictor) and weight, and weight-related behaviours.

**Results:**

Higher perceived stress in women was associated with a higher BMI, and to increased odds of being obese in cross-sectional and longitudinal analyses. Cross-sectional and longitudinal associations were found between stress and both less leisure-time physical activity, and more frequent fast food consumption. Longitudinal associations were also found between stress and increased television viewing time.

**Conclusion:**

The present study contributes to the literature related to the effects of stress on weight and weight-related behaviours. The findings suggest that higher stress levels could contribute to obesity risk in women. Further research is needed to fully understand the mechanisms underlying these associations. However, interventions that incorporate stress management techniques might help to prevent rising obesity rates among socioeconomically disadvantaged women.

## Background

Current rates of overweight and obesity in developed countries present a major threat for population health [1]. Obesity is a significant risk factor for a range of adverse health conditions, including type 2 diabetes, stroke, cardiovascular disease and various forms of cancer [2]. Certain population groups are at increased risk of overweight and obesity, with higher levels of obesity reported in women of childbearing age [3,4], those who are socioeconomically disadvantaged or those who are living in socioeconomically disadvantaged neighbourhoods [5-8]. While poor diets and physical inactivity are recognised as key behaviours implicated in the aetiology of obesity [9-12], the determinants of the increased risk of obesity and its determinant behaviours amongst socioeconomically disadvantaged groups remain poorly understood.

One key factor suggested to be linked to the development of obesity and which may be particularly pertinent among socioeconomically disadvantaged groups is psychological stress. Several studies have reported that indicators of chronic stress are associated with greater abdominal adiposity [13,14]. A systematic review of the literature reported less healthy eating patterns and higher body weight in individuals in lower social positions who had higher stress levels, with these patterns more apparent in women than men [15]. A meta-analysis of longitudinal studies showed that stress was associated with increasing adiposity [16]. Furthermore, higher levels of stress in the family reportedly increases children’s obesity risk [17,18], and several studies have reported associations between work stress and obesity risk [19-21]. For example, work stress has been associated with increased body mass index (BMI) at follow-up in a group of male and female employees, with findings also showing increased alcohol consumption and decreased vegetable consumption in workers with low job control [22]. However, research on these relationships has produced inconsistent results [23,24]. For example, in a group of low-income young mothers perceived stress was not a significant predictor of obesity [23].

There are few longitudinal studies that have explored the relationships between stress, body weight and weight-related behaviours. Longitudinal studies can provide insights into the direction and potential nature of associations among these variables. It is plausible that obesity is a consequence of stress, for example reflecting the use of maladaptive coping strategies such as comfort eating or excessive sedentary behaviours [25]. Previous studies have reported that chronic stress is associated with binge or comfort type eating [26], reduced physical activity levels [27] and increased sedentary behaviours [28]. Preferences for more palatable, higher fat, energy dense foods have also been associated with stress [29,30]. However, prospective research is limited, and confirmation of the temporal nature of these associations in longitudinal studies is required. Furthermore, few studies have explored these relationships in socioeconomically disadvantaged women. As living in a socioeconomically disadvantaged neighbourhood places residents at increased risk of both obesity [31] and psychological stress [32], examining associations between these factors is particularly pertinent in this vulnerable population. The aim of this study was to determine whether perceived stress was associated cross-sectionally and longitudinally with weight and weight-related behaviours in a cohort of women living in socioeconomically disadvantaged neighbourhoods.

## Methods

### Sample

This study examined baseline (T1) and three-year follow-up (T2) data collected in 2007–08 and 2010–2011 as part of the Resilience for Eating and Activity Despite Inequality (READI) study [33]. This multilevel study followed a cohort of women aged 18 to 46 years living in socioeconomically disadvantaged neighbourhoods. Ethical approval for the study was given by the Deakin University Human Research Ethics Committee, the Victorian Department of Education and the Catholic Education Office.

Forty rural and 40 urban neighbourhoods (suburbs) were randomly selected from the most socioeconomically disadvantaged third of all areas across Victoria, Australia, according to the Australian Bureau of Statistics’ (ABS) Socioeconomic Index for Areas [34]. The sampling framework only included neighbourhoods with more than 1200 inhabitants and within 200 km from Melbourne.

One hundred fifty (150) women from each of the 80 neighbourhoods were randomly selected from the electoral roll. As voting is compulsory for Australian adults, the electoral roll provides a relatively complete record of population data in Australian residents aged 18 years and over. Where there were fewer than 150 women living in the neighbourhood (n = 3 neighbourhoods), all those who were eligible were invited to participate. A T1 self-report survey was mailed to an initial sample of 11940 women between August 2007 and January 2008. The survey assessed women’s physical activity, eating behaviours, height and weight, and a broad range of factors thought to influence these behaviours and obesity risk. A reminder protocol [35] was employed whereby letters were sent to nonresponders 10 days after the initial survey package was mailed. A second reminder letter followed including another copy of the survey after a further 10 days. The surveys were initially pilot-tested with a convenience sample of 32 women aged 18 to 46 years and minor modifications were made for clarity based on the feedback received.

A total of 4934 women returned a completed survey. Excluding those surveys marked ‘return to sender’ (n = 861) or from women who were otherwise ineligible (e.g., were deceased, or were incorrectly denoted as females on the electoral roll); this represented a response rate of 45%. Data from a further 571 women were excluded because the women no longer lived in a READI neighbourhood, nine were excluded because they were not within the desired age range (18 to 46 years), three were excluded because the survey was not completed by the woman it was addressed to, and two subsequently requested to be withdrawn from the study. This left a total of 4349 women with T1 data. Comparison of the T1 READI sample with the general population of women living in the 80 neighbourhoods recorded in the 2006 Census [36,37] showed that a greater proportion of READI women were Australian born (89% vs. 73%), and were married or living as married (65% vs. 49%), but a lower proportion of READI women were in full-time employment (37% vs. 58%).

Three years following the T1 survey, all participants who consented to further follow-up in their T1 survey and remained in a READI neighbourhood (n = 2850) were sent a T2 survey, which repeated most of the questions in the T1 survey. Fifty-one women were excluded as they moved out of a READI neighbourhood. One thousand nine hundred twelve T2 surveys (n = 1912) were returned. Data from 483 women were excluded due to missing outcome data at T1 or T2, 81 missing covariate data and 8 missing stress scores. Some women had missing data on more than one set of variables, leaving an analysis sample of 1382.

### Measures

#### Sociodemographic characteristics

Participants were asked to provide sociodemographic information including age, highest level of education (categorised as ‘low’ - did not complete high school, ‘medium’ - completed high school/trade certificate/diploma, or ‘high’ - completed tertiary education), marital status (categorised as ‘married’ - married/de facto, ‘previously married’ - separated/divorced/widowed, or ‘never married’), employment status (categorised as ‘working full-time’, ‘working part-time’ or ‘not currently employed in paid work’), smoking status (categorised as ‘never smoked’, ‘used to smoke’, ‘smoke occasionally’, or ‘smoke regularly’), country of birth (categorised as either ‘Australia’ or ‘other’), serious illness, long term injury or disability that prevents physical activity (categorised as ‘yes’ or ‘no’) and the number of dependent children (categorised as ‘none’, ‘one’, ‘two’, or ‘three or more’).

#### Weight and BMI

Participants reported their height at T1 and weight at T1 and T2. BMI was calculated for each participant at T1 and T2 by dividing weight (in kilograms) by height (in metres) squared, and categorised as healthy weight (18.5–24.9 kg m^-2^), overweight (25.0–29.9 kg m^-2^) or obese (BMI 30.0 kg m^-2^ or more) [2]. Due to the very low number of women in the underweight category (BMI <18.5 kg m^-2^) (n = 126), data for these women were combined with those in the healthy weight category.

#### Physical activity and sedentary behaviours

Physical activity at T1 and T2 was assessed using the long version of the self-administered International Physical Activity Questionnaire (IPAQ-L), a well-established survey with demonstrated test-retest reliability and validity [38]. The IPAQ-L was used to measure leisure-time physical activity (LTPA) and the amount of time women spent sitting in the last seven days. Women were also asked to report the amount of time spent sitting watching television during the past week on both weekdays and weekend days. For each of the T1 and T2 physical activity and sedentary behaviour measures, tertile splits were used to categorise women as spending ‘low’, ‘medium’ or ‘high’ amount of time engaged in the activity. Tertile splits were used due to highly skewed distributions in the data.

#### Food habits

Six variables were used as indicators of food habits. These were selected based on their high energy/low nutrient content and they are likely important contributors to high-energy intake and obesity risk [39,40]. They include; potato crisps or salty snack food; chocolate or lollies; cake, doughnuts or sweet biscuits; pies, pasties or sausage rolls; fast foods; pizza; and non-diet soft drink. These variables were assessed using a Food Frequency Questionnaire (FFQ), which was based on several previously published and validated Australian questionnaires and assessed food habits during the previous month [41-43]. There were nine response categories for each food item ranging from ‘Never or less than once/month’ to ‘6 or more times a day’. For the soft drink item, six response options ranged from ‘I don’t drink soft drink’ to ‘10 or more serves/day’. For each of the T1 and T2 food habits measures, tertile splits based on the distributions were used to categorise women as having ‘low’, ‘medium’ or ‘high’ intake of each food or drink item.

#### Perceived stress

Stress was measured using the 4-item Perceived Stress Scale (PSS), which is used to measure the extent to which individuals consider situations in their life as stressful in terms of feeling in control [44]. The PSS has previously shown adequate reliability and validity among a sample of males and females participating in a smoking cessation program [45]. The specific questions were: “During the last month how often have you: (i) felt that you were unable to control the important things in your life? (ii) felt confident about your ability to handle your personal problems? (iii) felt that things were going your way? (iv) felt difficulties were piling up so high that you could not overcome them?” Responses were categorised as ‘never’ (scored 1), ‘almost never’ (2), ‘sometimes’ (3), ‘fairy often’ (4), or ‘very often’ (5). Perceived stress scores were then calculated by reverse scoring the positively stated items (ii and iii) and then summing all scale items (Cronbach’s alpha = 0.78).

### Statistical methods

The data were analysed using SPSS Statistics 18.0 (SPSS Inc., Chicago, IL, USA) and STATA Version 12 (Stata-Corp, College Station, TX). Multinomial logistic regression (MLR) was used to examine the cross-sectional associations between perceived stress, weight-related behaviours and weight status. Additionally, a linear regression model was tested to examine the cross-sectional association between perceived stress and continuous BMI. Separate models were analysed for each outcome measure. T1 perceived stress was the predictor.

Longitudinal associations between perceived stress, weight-related behaviours and weight status outcomes were examined using MLR. A linear regression model was used to examine the longitudinal association between perceived stress and continuous BMI. Separate longitudinal models were tested for each of the T2 weight and weight-related behaviour outcomes, and included T1 perceived stress as the predictor. Each longitudinal model also controlled for the corresponding T1 weight or behavioural measure, e.g. in the model with T2 soft drink intake as an outcome, T1 soft drink intake was included as a covariate. In each MLR analysis, ‘low’ was the reference category for weight-related outcomes. All analyses controlled for age, education level, marital status, employment status, smoking status, the number of dependent children and country of birth, all assessed at T1. All models were also adjusted for clustering by neighbourhood. The presence of a serious illness, long term injury or disability that prevents physical activity was also controlled for in all regression analyses predicting physical activity and sedentary behaviour.

## Results

T1 sociodemographic characteristics of the sample are presented in Table 1. The mean age of the sample was 35.71 years (s.d. = 7.7) and the mean BMI was 26.2 (s.d. = 5.9). Most of the women were born in Australia (92.3%) and had a medium level of education (49.7%).

**Table 1:** T1 (baseline) characteristics of the READI sample (n = 1382)

	**Mean**	**SD**
Age (years)	35.7	7.7
BMI	26.2	5.9
Stress	10.0	2.8
	**n**	**%**
*Education*		
Low – did not complete high school	274	19.8
Medium – completed high school/trade certificate/diploma	687	49.7
High – completed tertiary education	421	30.5
*Marital status*		
Married/defacto relationship	1002	72.5
Separated/divorced/widowed	103	7.5
Never married	277	20.0
*Number of children (aged up to 18 years living with woman)*		
None	515	37.3
One	244	17.7
Two	380	27.5
Three or more	243	17.6
*Employment status*		
Working full-time	533	38.6
Working part-time	440	31.8
Not currently employed (paid work)	409	29.6
*Country of birth*		
Not Australia	107	7.7
Australia	1275	92.3
*Serious illness, long term injury or disability that prevents physical activity*		
Yes	139	10.1
No	1243	89.9
*Smoking status*		
Never smoked	730	52.8
Used to smoke	359	26.0
Smoke occasionally	114	8.2
Smoke regularly	179	13.0

Table 2 shows the distributions of outcomes within the sample at T1 and T2. At T1, over half of the women were in the healthy weight range (52.2%), 26.8% overweight and 20.9% obese. Forty-seven percent of women were in the healthy weight range at T2, 29.7% overweight and 23.6% obese. Thirty-one percent of women engaged in 52 or fewer minutes of LTPA per week (the cut point for the lowest tertile of LTPA) at T1, and 32% at T2. Thirty-four percent engaged in more than 52 hours of sitting time per week, and 34% watched between 14–21 hours of television per week at T1. Similarly, 33% engaged in more than 52 hours of sitting time per week, and 35% watched between 14–21 hours of television per week at T2. Potato crisps or salty snack food intake of once or more times per week was reported by 42% of the sample at T1. However, 42% of the sample reported potato crisps or salty snack food intake 1–3 times per month at T2. Most women consumed chocolates or lollies twice or more times per week at T1 and T2. Fifty-one percent and 56% of women reported that they did not drink soft drink (excluding diet soft drink) at T1 and T2, respectively. Most women reported consuming cake, doughnuts and sweet biscuits, and pizza less than once per week, and pies, pastries or sausage rolls, and consuming fast foods (e.g. McDonalds, KFC) less than monthly at T1 and T2.

**Table 2:** Distributions of outcomes within READI sample at T1 and T2

		**T1**		**T2**	
		**n**	**%**	**n**	**%**
BMI category					
	Healthy weight (18.5–24.9 kg m-^2^)	722	52.2	645	46.7
	Overweight (25.0–29.9 kg m^-2^)	371	26.8	411	29.7
	Obese (BMI 30.0 kg m^-2^ or more)	289	20.9	326	23.6
LTPA (per week)					
	Low (≤52 mins)	423	30.6	444	32.1
	Medium (53 mins-4 hours)	499	36.1	482	34.9
	High (5+ hours)	460	33.3	456	33.0
Sitting time (per week)					
	Low (≤30 hours)	460	33.3	461	33.4
	Medium (31–52 hours)	457	33.1	460	33.3
	High (53+ hours)	465	33.6	461	33.4
Television viewing time (per week)					
	Low (≤13 hours)	459	33.2	460	33.3
	Medium (14–21 hours)	474	34.3	487	35.2
	High (22+ hours)	449	32.5	435	31.5
Potato crisps or salty snack foods					
	Low (<once/month)	247	17.9	248	17.9
	Medium (1–3 times/month)	555	40.2	575	41.6
	High (1+ times/week)	580	42.0	559	40.4
Chocolates or lollies					
	Low (≤3 times/month)	392	28.4	418	30.2
	Medium (once/week)	363	26.3	356	25.8
	High (2+ times/week)	627	45.4	608	44.0
Cake, doughnuts and sweet biscuits					
	Low (≤3 times/month)	622	45.0	662	47.9
	Medium (once/week)	357	25.8	348	25.2
	High (2+ times/week)	403	29.2	372	26.9
Pies, pastries or sausage rolls					
	Low (<once/month)	653	47.3	639	46.2
	Medium (1–3 times/month)	544	39.4	567	41.0
	High (1+ times/week)	185	13.4	176	12.7
Fast foods (e.g. McDonalds, KFC)					
	Low (<once/month)	629	45.5	615	44.5
	Medium (1–3 times/month)	499	36.1	545	39.4
	High (1+ times/week)	254	18.4	222	16.1
Pizza					
	Low (<once/month)	591	42.8	600	43.4
	Medium (1–3 times/month)	673	48.7	688	49.8
	High (1+ times/week)	118	8.5	94	6.8
Soft drink (excluding diet soft drink)					
	Low (don’t drink soft drink)	707	51.2	767	55.5
	Medium (<1 serve/day)	462	33.4	446	32.3
	High (1+ serves/day)	213	15.4	169	12.2

Table 3 shows the cross-sectional associations between stress and weight and behavioural outcomes at T1. A positive association was found between stress levels and continuous BMI (B = 0.28, CI = 0.17, 0.39, p < 0.0005). Furthermore, for every increase of one unit on the stress scale, there was an increase of 6% (CI = 1.01,1.11, p = 0.013) in the odds of being overweight, and 13% (CI = 1.08,1.19, p <0.0005) increase in the odds of being obese. Stress levels were associated with LTPA, such that higher stress scores were associated with a lower likelihood of undertaking medium (OR = 0.93, CI = 0.88,0.99, p = 0.014) or high (OR = 0.91, CI = 0.86,0.96, p < 0.0005) levels of LTPA. There were no cross-sectional associations found between stress and sedentary behaviour measures. Similarly, few cross-sectional associations between stress and measures of food habits were found. However, stress was associated with increased odds of high intakes of fast foods (OR = 1.09, CI = 1.02,1.17, p = 0.010).

**Table 3:** MLR analyses of cross-sectional associations between stress and behavioural outcomes at T1

**T1 outcomes**^**a**^		**β**^**b**^	**B**^**c**^	**(95% CI)**	**P**
**BMI**		**0.13**	**0.28**	**(0.17, 0.39)**	**<0.0005**
			**OR**	**(95% CI)**	**P**
BMI category					
	Healthy weight (18.5–24.9 kg m-^2^)				
	**Overweight (25.0–29.9 kg m**^**-2**^**)**		**1.06**	**(1.01,1.11)**	**0.013**
	**Obese (BMI 30.0 kg m**^**-2 **^**or more)**		**1.13**	**(1.08,1.19)**	**<0.0005**
LTPA (per week)					
	Low (≤52 mins)				
	**Medium (53 mins-4 hours)**		**0.93**	**(0.88,0.99)**	**0.014**
	**High (5+ hours)**		**0.91**	**(0.86,0.96)**	**<0.0005**
Sitting time (per week)					
	Low (≤30 hours)				
	Medium (31–52 hours)		1.01	(0.97,1.06)	0.593
	High (53+ hours)		0.99	(0.95,1.03)	0.622
Television viewing time (per week)					
	Low (≤13 hours)				
	Medium (14–21 hours)		1.02	(0.97,1.07)	0.447
	High (22+ hours)		0.99	(0.94,1.03)	0.544
Potato crisps or salty snack foods					
	Low (<once/month)				
	Medium (1–3 times/month)		1.02	(0.96,1.08)	0.495
	High (1+ times/week)		1.05	(0.98,1.11)	0.145
Chocolates or lollies					
	Low (≤3 times/month)				
	Medium (once/week)		0.99	(0.94,1.05)	0.795
	High (2+ times/week)		1.04	(0.99,1.09)	0.161
Cake, doughnuts and sweet biscuits					
	Low (≤3 times/month)				
	Medium (once/week)		1.00	(0.96,1.04)	0.954
	High (2+ times/week)		1.00	(0.96,1.05)	0.890
Pies, pastries or sausage rolls					
	Low (<once/month)				
	Medium (1–3 times/month)		0.97	(0.93,1.01)	0.114
	High (1+ times/week)		1.00	(0.93,1.07)	0.953
Fast foods (e.g. McDonalds, KFC)					
	Low (<once/month)				
	Medium (1–3 times/month)		1.04	(1.00,1.09)	0.069
	**High (1+ times/week)**		**1.09**	**(1.02,1.17)**	**0.010**
Pizza					
	Low (<once/month)				
	Medium (1–3 times/month)		0.97	(0.92,1.02)	0.196
	High (1+ times/week)		1.01	(0.94,1.08)	0.817
Soft drink (excluding diet soft drink)					
	Low (don’t drink soft drink)				
	Medium (<1 serve/day)		1.00	(0.95,1.04)	0.930
	High (1+ serves/day)		1.07	(1.00,1.14)	0.053

^a^‘Low’ is the reference category for all outcomes.

Note: All analyses controlled for age, education level, marital status, employment status, smoking status, the number of dependent children, country of birth and clustering by neighbourhood. Bolded associations were significant.

^b^Standardised regression coefficient.

^c^Unstandardised regression coefficient with 95% confidence interval.

Table 4 shows the longitudinal associations between stress (T1) and weight and behavioural outcomes (T2). A positive association was found between stress levels and BMI (B = 0.085, CI = 0.04-0.13, p < 0.0005). Stress was not predictive of being in the overweight BMI category (25.0–29.9 kg m^-2^), but higher stress levels were associated with an increase of 11% (CI = 1.00,1.23, p = 0.043) in the odds of being obese at T2. Longitudinal associations were also found between stress levels and the likelihood of engaging in medium (OR = 0.93, CI = 0.88, 0.98, p = 0.004) or high (OR = 0.89, CI = 0.84,0.94, p <0.0005) amounts of LTPA. Associations were also found between stress and television viewing, such that each increase of one unit corresponded to a 7% increase in the odds of watching medium amounts of television (CI = 1.01,1.12, p = 0.014). Consistent with cross-sectional associations, stress and fast food intake were longitudinally associated, such that greater stress scores predicted increased likelihood of consuming high intake of fast food (OR = 1.08, CI = 1.02,1.14, p = 0.011), but there were no other dietary associations.

**Table 4:** MLR analyses of longitudinal associations between T1 stress and behavioural outcomes at T2

**T2 outcomes**^**a**^		**β**^**b**^	**B**^**c**^	**(95% CI)**	**P**
BMI		**0.04**	**0.09**	**(0.04, 0.13)**	**<0.0005**
			**OR**	**(95% CI)**	**P**
BMI category					
	Healthy weight (18.5–24.9 kg m-^2^)				
	Overweight (25.0–29.9 kg m^-2^)		1.02	(0.97,1.08)	0.454
	**Obese (BMI 30.0 kg m**^**-2 **^**or more)**		**1.11**	**(1.00,1.23)**	**0.043**
LTPA (per week)					
	Low (≤52 mins)				
	**Medium (53 mins-4 hours)**		**0.93**	**(0.88,0.98)**	**0.004**
	**High (5+ hours)**		**0.89**	**(0.84,0.94)**	**<0.0005**
Sitting time (per week)					
	Low (≤30 hours)				
	Medium (31–52 hours)		0.96	(0.92,1.00)	0.051
	High (53+ hours)		1.02	(0.97,1.08)	0.339
Television viewing time (per week)					
	Low (≤13 hours)				
	**Medium (14–21 hours)**		**1.07**	**(1.01,1.12)**	**0.014**
	High (22+ hours)		1.04	(0.98,1.11)	0.146
Potato crisps or salty snack foods					
	Low (<once/month)				
	Medium (1–3 times/month)		1.01	(0.95,1.07)	0.745
	High (1+ times/week)		1.04	(0.98,1.10)	0.219
Chocolates or lollies					
	Low (≤3 times/month)				
	Medium (once/week)		1.01	(0.96,1.07)	0.652
	High (2+ times/week)		1.02	(0.97,1.07)	0.453
Cake, doughnuts and sweet biscuits					
	Low (≤3 times/month)				
	Medium (once/week)		1.01	(0.96,1.05)	0.787
	High (2+ times/week)		1.00	(0.95,1.04)	0.931
Pies, pastries or sausage rolls					
	Low (<once/month)				
	Medium (1–3 times/month)		0.98	(0.94,1.02)	0.401
	High (1+ times/week)		1.05	(0.98,1.12)	0.154
Fast foods (e.g. McDonalds, KFC)					
	Low (<once/month)				
	Medium (1–3 times/month)		1.04	(0.99,1.08)	0.103
	**High (1+ times/week)**		**1.08**	**(1.02,1.14)**	**0.011**
Pizza					
	Low (<once/month)				
	Medium (1–3 times/month)		0.98	(0.94,1.03)	0.483
	High (1+ times/week)		1.02	(0.94,1.11)	0.628
Soft drink (excluding diet soft drink)					
	Low (don’t drink soft drink)				
	Medium (<1 serve/day)		0.98	(0.93,1.03)	0.378
	High (1+ serves/day)		1.04	(0.95,1.15)	0.398

^a^‘Low’ is the reference category for all outcomes.

Note: All analyses controlled for age, education level, marital status, employment status, smoking status, the number of dependent children, country of birth and clustering by neighbourhood. Bolded associations were significant.

^b^Standardised regression coefficient.

^c^Unstandardised regression coefficient with 95% confidence interval.

## Discussion

This study examined the associations between stress, weight and weight-related behaviours in a cohort of women living in socioeconomically disadvantaged neighbourhoods. The findings of this study are generally consistent with those of similar studies in other populations regarding the relationships between stress and weight [15,19]. The present study found that higher stress in women was associated with increased odds of having a higher BMI, and of being obese. Cross-sectional and longitudinal associations were found between stress and both less leisure-time physical activity, and more frequent fast food consumption. Longitudinally, stress was also found to be a predictor of increased television viewing time.

Consistent with our findings, several studies have reported associations between stress and BMI [15,19,46]. A meta-analysis of longitudinal studies on stress and adiposity also found that stress promotes weight gain [16]. However, a study of adolescents in the United Kingdom found that there was no association between perceived stress and increases in weight over five years [24]. Inconsistencies in these results may be due to the difficulty in measuring stress, particularly in different age groups and populations. In the study with adolescents, the PSS was used to measure stress. This measure was initially developed to subjectively measure stress in adults, and adolescents may interpret questions differently. More valid measures of stress might provide more consistency in results among different studies. The majority of previous studies of stress and weight have not assessed associated weight-related behaviours, and hence shed little insight into potential mechanisms by which stress may influence weight change or obesity risk. Existing studies on the associations between stress and physical activity, for example, have produced inconsistent results [27,47,48]. However, our findings concur with those of several past studies showing that stress is associated with engaging in less physical activity [27,47]. Less engagement in physical activity due to stress may reflect challenging life circumstances and difficulty coping, which may take precedence over self-care and health-promoting behaviours like physical activity [48]. Furthermore, despite evidence of the beneficial effects of physical activity on stress [49], many individuals may find sedentary activity more rewarding in the short-term [50].

Evidence of the associations between stress and sedentary behaviours are limited. The present study found that stress was longitudinally associated with moderate amounts of television viewing. Similarly, past research has shown that highly stressed parents of ill children were found to watch more hours of television than parents of healthy children [46]. Individuals in situations of high stress are more likely to engage in unhealthy behaviours that make them feel better [51]. Therefore, stressed women may seek comfort from television viewing or use television as a distraction from stressful thoughts.

The present study reported few associations between stress and measures of food habits. This may be due to the use of the FFQ in our study, which did not assess portion size, and hence may not be a sufficiently sensitive instrument to detect any associations between consumption of larger quantities of such food items and stress. However, stress was found to predict higher intakes of fast food consumption. Consistent with our findings, a study by Bauer et al. [17] used a series of questions to measure the frequency of fast food consumption in parents and reported more frequent fast food consumption in parents with greater work-life stress. It is possible that disadvantaged women who are feeling stressed may turn to fast food as a perceived ‘quick fix’, for instance if they are time poor; or it could be that the types of foods typically purchased in fast food outlets are perceived as ‘comfort’ or ‘reward’ foods and used to cope with stress [52]. A diary study assessing daily food choice of 30 food items reported that higher intake of soft drinks and lollies, particularly chocolate have been reportedly associated with stress [48], but this was not found in the present study. These discrepant findings may be attributable to the different food intake measures used, with a diary study potentially capturing daily variances in consumption more readily than the FFQ used in our study.

This study had a number of strengths. These include analyses of a large sample from a population of women living in socioeconomically disadvantaged neighbourhoods and who are at high risk of weight gain. The large sample size also allowed control for a range of key covariates. Furthermore, this study is one of few longitudinal studies assessing the relationships between stress, weight and weight-related behaviours. Limitations of this study include the reliance on self-report data, although established and validated measures were used where possible (e.g., the IPAQ-L to measure physical activity). Height and weight were self-reported which may have led to an underestimate of prevalence of overweight and obesity. This may have resulted in misestimation of the strength of associations between stress and overweight and obesity. However, recent evidence suggests substantial agreement between self-report and measured height and weight among Australian women [53]. Food habits were assessed with only a selected subset of FFQ items, and while these were based on previously validated scales, the validity of this subset of questions alone is not established. There was a modest response rate to the survey, and considerable loss to follow-up. For example, longitudinal analyses in this study were based on a sample of which approximately 10% were originally sampled. Since we have no information on weight status or stress from non-respondents to the initial mailout, we cannot conclude how this bias may affect results. However, such response and attrition are not atypical for this population [54,55]. It should also be acknowledged that associations between stress and weight could operate in the reverse direction to that tested in the present study. That is, weight gain and obesity may lead to increased stress, for instance due to weight-related stigmatization or poor physical or mental health associated with obesity. Consistent with this hypothesis, several studies have shown an association between obesity and future symptoms of depression [56-58]. This remains a question for future research.

Acknowledging the study’s limitations, and the need for further confirmation of the mechanisms underlying the associations observed here, the findings from this study have important implications for public health practice, suggesting a potential key role for psychological stress in weight and weight-related behaviours. Public health interventions might benefit from the inclusion of stress management in weight loss interventions to address psychological health and maximise individuals’ weight loss and weight maintenance attempts. Particularly, the role of physical activity in reducing stress (and weight) could be emphasised in specifically targeted programs.

## Conclusion

In conclusion, the present study demonstrated some cross-sectional and longitudinal associations between perceived psychological stress and BMI, as well as leisure-time physical activity, sedentary behaviour and fast food consumption. Developing intervention strategies to improve coping skills during situations of stress might assist women in socioeconomically disadvantaged neighbourhoods to manage their weight more effectively.

## Abbreviations

READI: Resilience for eating and activity despite inequality; SIFA: Socioeconomic index for areas; PSS: Perceived stress scale; FFQ: Food frequency questionnaire; IPAQ: International physical activity questionnaire; LTPA: Leisure-time physical activity; MLR: Multinomial logistic regression.

## Competing interests

The authors declared that they have no competing interests.

## Authors’ contributions

JM carried out background research and drafted the manuscript. GA and KB assisted JM in performing statistical analysis and helped draft the manuscript. KB conceived the idea for and implemented the READI study, and developed the measures and methods. All authors read and approved the final manuscript.

## Pre-publication history

The pre-publication history for this paper can be accessed here:

http://www.biomedcentral.com/1471-2458/13/828/prepub
